# Effects of ApoE genotype on clinical phenotypes in early‐onset and late‐onset Alzheimer's disease in China: Data from the PUMCH dementia cohort

**DOI:** 10.1002/brb3.2373

**Published:** 2021-09-23

**Authors:** Liling Dong, Jie Li, Caiyan Liu, Chenhui Mao, Jie Wang, Dan Lei, Xinying Huang, Shanshan Chu, Bo Hou, Feng Feng, Longze Sha, Qi Xu, Jing Gao

**Affiliations:** ^1^ Neurology Department State Key Laboratory of Complex Severe and Rare Diseases Peking Union Medical College Hospital Chinese Academy of Medical Sciences and Peking Union Medical College Dongcheng Beijing China; ^2^ Department of Radiology State Key Laboratory of Complex Severe and Rare Diseases Peking Union Medical College Hospital Chinese Academy of Medical Sciences and Peking Union Medical College Dongcheng Beijing China; ^3^ Institute of Basic Medical Sciences Peking Union Medical College Dongcheng Beijing China

**Keywords:** ApoE, cognitive function, cortical atrophy, early‐onset Alzheimer's disease, late‐onset Alzheimer's disease, tau burden

## Abstract

**Introduction:**

To investigate the heterogeneous effect of Apolipoprotein E (ApoE) genotype on clinical phenotypes in early‐onset Alzheimer's disease (EOAD) and late‐onset Alzheimer's disease (LOAD), respectively.

**Methods:**

785 probable AD patients were enrolled from the dementia cohort of Peking Union Medical College Hospital (PUMCH), China. There were 386 EOAD and 399 LOAD cases. All individuals finished history inquiry, neurological examination, blood biochemical test, neuropsychological screening test, electroencephalography, brain CT/MRI, and ApoE genotyping. Some participants had neuropsychological domain assessment (*n* = 317), MRI morphometry (*n* = 130), CSF testing of Aβ42, p‐tau, t‐tau (*n* = 144), or DNA sequencing (*n* = 690). The variables were compared mainly between ɛ4 carriers and non‐carriers in EOAD and LOAD, respectively.

**Results:**

In LOAD, ɛ4 carriers showed female predominance; worse performance in trail making test, delayed recall of auditory verbal learning test (AVLT) and rey complex figure; smaller hippocampal, parahippocampal, and entorhinal volume, as compared to ɛ4 non‐carriers. In EOAD, ɛ4 carriers had lower scores in AVLT, episodic memory and modified Luria's tapping task; but less cortical atrophy in entorhinal, middle cingulate, inferior frontal, and parieto‐occipital regions, in comparison to ɛ4 non‐carriers. 6.2% (43/690) subjects harbored potential causative mutations in APP, PSEN1, and PSEN2. In both EOAD and LOAD, no differences were observed between ɛ4 carriers and non‐carriers in CSF levels of Aβ42, p‐tau, t‐tau, or mutation frequency.

**Conclusions:**

ApoE exerts a heterogeneous effect on clinical phenotypes in EOAD and LOAD, which might be related to the different genetic and pathological basis underlying them.

## Graphical abstract

ApoE exerts a heterogeneous effect on clinical phenotype in EOAD and LOAD. In LOAD, ApoE‐ɛ4 genotype is associated with severe medial temporal atrophy and a higher p‐tau level. However, in EOAD, ɛ4 genotype is associated with less cortical atrophy in widespread areas, and lower t‐tau level.

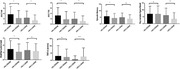

## 1 INTRODUCTION

It is well established that Apolipoprotein E (ApoE) ɛ4 genotype is a genetic risk factor for Alzheimer's disease (AD). ApoE‐ɛ4 allele might be involved in almost all AD pathological processes, including amyloid beta (Aβ) aggregation, neurofibrillary tangle formation, cholinergic activity, cholesterol metabolism, synaptic integrity, and plasticity (Kotze et al., 2015; Safieh et al., 2019).

Many studies focused on the relevance between ApoE and clinical phenotype in AD. However, the results were inconsistent. Several studies agreed that ApoE‐ε4 was associated with memory deficit or global cognitive impairment, whereas some stated that ApoE‐ε4 had little effect on cognitive function (Bondi et al., 2003; van der Vlies et al., 2007; Vivot et al., 2015). Some studies demonstrated that ApoE‐ε4 was associated with small hippocampal volume, whereas some did not (Liu et al., 2015).

The inconsistencies in these findings might be related to the heterogeneity of AD. Based on the age of onset (AOO), AD can be divided into early‐onset AD (EOAD, AOO < 65 years old) and late‐onset AD (LOAD, AOO ≥ 65 years old). EOAD and LOAD are heterogeneous in terms of genotype, phenotype and pathology. Pathogenic mutations in APP, PSEN1, and PSEN2 can be detected in 5–10% EOAD patients (Cacace et al., 2016). However, less than 1% of LOAD cases can be explained by these causative mutations (Cruchaga et al., 2012). Most LOAD patients are characterized by memory symptoms. About 25% EOAD cases present with atypical non‐memory symptoms, such as apraxia, aphasia, visual, or executive dysfunction (Bateman et al., 2011; Flier et al., 2011; Reitz et al., 2020; Ryan & Rossor, 2010). Compared with EOAD, LOAD subjects are more likely to have comorbid pathologies, such as TDP‐43, Lewy bodies or vascular pathology (Haroutunian et al., 2008; Middleton et al., 2011; Reitz et al., 2020; Savva et al., 2009).

Considering the heterogeneity between EOAD and LOAD, we hypothesized that ApoE genotype exerted a heterogeneous effect on clinical phenotype in EOAD and LOAD. First, we compared the clinical phenotype between EOAD and LOAD to further confirm their intrinsic heterogeneity. Second, we illustrated the heterogeneous effect of ApoE on clinical phenotype in EOAD and LOAD, respectively. We aimed to have a comprehensive study of the relevance between ApoE and demographics, neuropsychology, neuroimaging, cerebrospinal fluid (CSF) biomarker, pathogenic variant, to better understand the mechanism of ApoE in AD.

## 2 METHODS

### 2.1 Participant

785 participants from the dementia cohort of Peking Union Medical College Hospital (PUMCH), China, were recruited between 2007 and 2019. All the participants were probable AD according to 2011 diagnostic guidelines for AD from National Institute on Aging‐Alzheimer's Association workgroups (McKhann et al., 2011).

This study was approved by the local ethics committee. Written informed consent was obtained. All individuals were required to finish history inquiry, neurological examination, blood biochemical test, neuropsychological screening test, electroencephalography, brain CT/MRI, and ApoE genotyping.

The participants voluntarily took neuropsychological domain assessment (*n* = 317), MRI morphometry (*n* = 130), FDG‐PET (*n* = 40), DNA sequencing (*n* = 690), or CSF testing of Aβ42, phosphorylated tau 181 (p‐tau), total tau (t‐tau) (*n* = 144).

### 2.2 Neuropsychological assessment

The screening test consisted of a mini‐mental state exam, Montreal cognitive assessment (PUMCH edition) (Tan et al., 2015), activities of daily living, hospital anxiety, and depression scale.

Domain assessment covered executive, visuospatial, memory, linguistic, and reasoning domains. It consisted of word fluency (Feiberg & Farah, 2003); digital symbol substitution task, similarity, calculation (Gong, 1982); graphics copying, block design (Gao, 1993); clock drawing, single gesture imitation, modified Luria's tapping task, Rey complex figure; trail making test part A (TMT‐A) (Gong, 1986); episodic memory (Gong, 1989), auditory verbal learning test (AVLT) (Guo et al., 2007), paired associate learning (The cooperation group for the construction of “The Clinical Memory Test,” 1986); as well as oral comprehension, repetition, naming and spontaneous speech (Gao, 1993).

### 2.3 MRI morphometry analysis

MRI scan was performed on the same scanner (Discovery MR750, GE, Milwaukee, Wisconsin). 3D structural imaging was acquired by 3D fast spoiled gradient echo sequence (BRAVO, 1 mm × 1 mm × 1 mm isotropic, Prep Time = 400 ms, bandwidth = 27.78 Hz, flip angle = 12°). Conventional MRI sequences included T1WI, T2WI, FLAIR, SWAN, and DWI.

The subjects with obvious intracranial lesions, such as hemorrhage, infarct, cyst, or tumor, would be excluded. Morphometry analysis was achieved by a range of SPM 12 techniques. First, the original images were spatially normalized by registering. After segmentation into gray matter, white matter, and CSF, the images were smoothed. Finally, a mapping template was created for each individual.

### 2.4 CSF testing

CSF samples were collected with 1.5 ml low protein binding tubes (Eppendorf LoBind, Hamburg, Germany) and centrifuged for 10 min at 1800 rpm, 4℃. The supernatant was stored at −80℃. CSF biomarkers (Aβ42, p‐tau and t‐tau) were determined by ELISA kits (INNOTEST β‐AMYLOID (1‐42), PHOSPHO‐TAU, hTAU Ag; Fujirebio, Ghent, Belgium).

### 2.5 DNA sequencing

The DNA library was sequenced on NextSeq500 sequencer (Illumina HiSeq X Ten Analyzers, San Diego, USA). All reads were aligned to the human reference genome (UCSC hg19). Variants were annotated using Annovar (version 2016Feb01) (Wang et al., 2010), referring to the guidelines from American College of Medical Genetics and Genomics (Richards et al., 2015).

### 2.6 Statistical analysis

The variables were compared between EOAD and LOAD, as well as between ɛ4 carriers and non‐carriers in EOAD and LOAD, respectively. The categorical variables were tested with Chi‐square test or Fisher's exact test. The continuous variables were tested with Student's *t*‐test. The neuropsychological, neuroimaging and CSF data were compared by the general linear model. Gender, age, education, and disease course were included in the model as fixed factors or covariates.

## 3 RESULTS

### 3.1 ApoE genotype (Table 1)

In all, there were 386 EOAD and 399 LOAD cases. Among EOAD subgroup, there were 235 ApoE‐ɛ4 non‐carriers and 151 ApoE‐ɛ4 carriers. Among LOAD subgroup, there were 210 ɛ4 non‐carriers and 189 ɛ4 carriers.

**TABLE 1:** ApoE distribution

	ε2ε2	ε2ε3	ε3ε3	ε2ε4	ε3ε4	ε4ε4	ε4 non‐carrier	ε4 carrier
	(*n* = 2)	(*n* = 56)	(*n* = 387)	(*n* = 15)	(*n *= 256)	(*n* = 69)	(*n* = 445)	(*n* = 340)
EOAD (*n* = 386)	1 (0.3%)	29 (7.5%)	205 (53.1%)	7 (1.8%)	108 (28.0%)	36 (9.3%)	235 (60.9%)	151 (39.1%)
LOAD (*n* = 399)	1 (0.3%)	27 (6.8%)	182 (45.6%)	8 (2.0%)	148 (37.1%)	33 (8.3%)	210 (52.6%)	189 (47.4%)
*p*	.139		.020*					

Abbreviations: EOAD, early‐onset Alzheimer's disease; LOAD, late‐onset Alzheimer's disease.

*P < 0.05.

LOAD patients had a higher ɛ4 allele frequency than EOAD patients (27.8% versus 24.2%, *p* = .123). The proportion of ApoE‐ɛ4 carriers was significantly higher in LOAD relative to EOAD (47.4% versus 39.1%, *p*  = .020).

### 3.2 Demographics (Table 2)

EOAD and LOAD did not differ in gender, education, and family history of dementia. EOAD patients showed a greater disease course than LOAD patients (3.5 ± 2.5 versus 3.1 ± 2.0 years, *p*  = .006), indicating delayed clinic visits in EOAD relative to LOAD.

**TABLE 2:** Sociodemographic features (mean ± SD)

	AD (*n* = 785)			EOAD (*n* = 386)			LOAD (*n* = 399)		
	EOAD (*n* = 386)	LOAD (*n* = 399)	*p*	ε4 non‐carrier (*n* = 235)	ε4 carrier (*n* = 151)	*p*	ε4 non‐carrier (*n* = 210)	ε4 carrier (*n* = 189)	*p*
Gender (M/F)	152/234	154/245	.822	95/140	57/94	.599	101/109	53/136	<.001***
Age (years old)	59.7 ± 6.2	77.2 ± 5.7	<.001***	59.4 ± 6.2	60.2 ± 6.3	.215	77.4 ± 6.0	77.0 ± 5.3	.423
AOO (years old)	56.2 ± 5.9	74.2 ± 5.8	<.001***	56.0 ± 5.7	56.5 ± 6.2	.455	74.5 ± 6.0	73.8 ± 5.6	.192
Disease course (years)^a^	3.5 ± 2.5	3.1 ± 2.0	.006**	3.4 ± 2.6	3.7 ± 2.3	.180	2.9 ± 1.9	3.2 ± 2.0	.123
Education (years)	9.6 ± 4.3	10.3 ± 5.3	.065	9.9 ± 4.2	9.3 ± 4.5	.233	10.7 ± 5.3	9.8 ± 5.3	.101
Family history of dementia (+/−)	161/225	154/245	.374	93/142	68/83	.288	70/140	84/105	.023*

Abbreviations: AOO, age of onset; EOAD, early‐onset Alzheimer's disease; LOAD, late‐onset Alzheimer's disease; M/F, male/female.

^a^ Disease course, the time interval from the disease onset to the clinic visit, when the patient finishes the testing.

*P < 0.05 **P < 0.01 ***P < 0.001.

In LOAD, compared with ɛ4 non‐carriers, ɛ4 carriers showed a higher proportion of female gender (72.0% versus 51.9%, *p*  < .001), and a higher proportion of positive family history of dementia (44.4% versus 33.3%, *p*  = .023).

In EOAD, no demographic difference was found between ɛ4 carriers and non‐carriers.

### 3.3 Neuropsychology (Supporting Information 1 and 2)

No neuropsychological difference was observed between EOAD and LOAD. After stratification by ApoE‐ɛ4 status, among ɛ4 carriers, EOAD patients finished TMT‐A at a faster speed than LOAD patients (96.2 ± 45.1 versus 114.4 ± 56.9 seconds, *p*  = .028).

**FIGURE 1:**
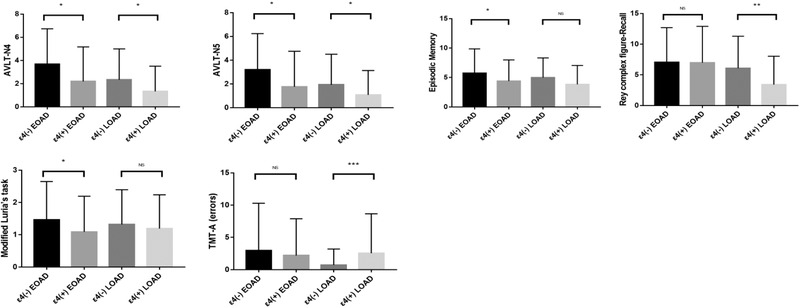
Comparison of neuropsychological features between ε4 carriers and non‐carriers in both EOAD and LOAD. The data were compared by general linear model. Gender, age, disease course, and educational level were included in the model as fixed factor or covariate. AVLT‐N4 and AVLT‐N5, short and long delayed recall of auditory verbal learning test; TMT‐A, trail making test part A

As was illustrated in Figure 1, in EOAD, ɛ4 carriers had lower scores in short and long delayed recall of AVLT (2.2 ± 3.0 versus 3.7 ± 3.0, *p*  = .014; 1.8 ± 3.0 versus 3.2 ± 3.0, *p*  = .041), episodic memory (4.4 ± 3.6 versus 5.7 ± 4.1, *p*  = .041), modified Luria's tapping task (1.1 ± 1.1 versus 1.5 ± 1.2, *p*  = .020) than ɛ4 non‐carriers. No difference was found between ɛ4 carriers and non‐carriers in neuropsychological screening tests.

In LOAD, ɛ4 carriers showed lower scores in short and long delayed recall of AVLT (1.3 ± 2.2 versus 2.3 ± 2.7, *p*  = .014; 1.1 ± 2.1 versus 1.9 ± 2.6, *p*  = .024), as well as delayed recall of rey complex figure (3.4 ± 4.6 versus 6.1 ± 5.2, *p*  = .004), in comparison to non‐carriers. ɛ4 carriers made more errors in TMT‐A than non‐carriers (2.5 ± 6.1 versus 0.7 ± 2.5, *p*  = .001). However, they showed no difference in the task completion time of TMT‐A (114.4 ± 56.9 versus 123.9 ± 71.0 seconds, *p* = .433). No difference was found between ɛ4 carriers and non‐carriers in neuropsychological screening tests.

Compared with the first immediate recall of AVLT, the third recall improved significantly in both EOAD (5.1 ± 2.5 versus 3.1 ± 1.7, *p*  < .001) and LOAD (4.8 ± 2.1 versus 2.8 ± 1.5, *p*  < .001). However, the improvement did not differ between ɛ4 carriers and non‐carriers.

### 3.4 Neuroimaging (Supporting Information 3)

The adjusted means showed that EOAD patients had smaller cortical volume (mm^3^) than LOAD patients in left occipital areas, including left middle occipital (4572 ± 1057 versus 4818 ± 673, *p*  = .020) and left inferior occipital gyrus (4604 ± 1047 versus 4865 ± 806, *p*  = .023). After stratification by ApoE‐ɛ4 status, among ɛ4 non‐carriers, EOAD patients had smaller cortical volume than LOAD patients, mainly in left parieto‐occipital and posterior cingulate regions.

**FIGURE 2:**
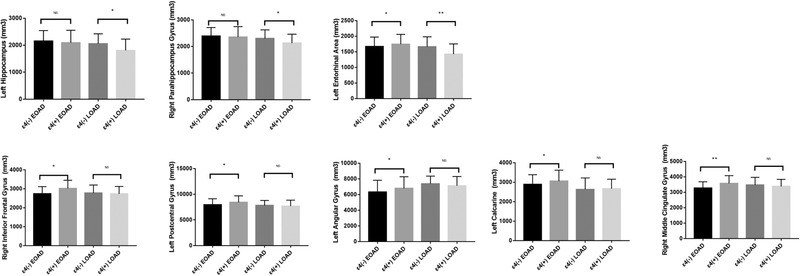
Comparison of MRI morphometric features between ε4 carriers and non‐carriers in both LOAD and EOAD. The data were compared by general linear model. Gender, age, disease course, and total intracranial volume were included in the model as fixed factor or covariate

**FIGURE 3:**
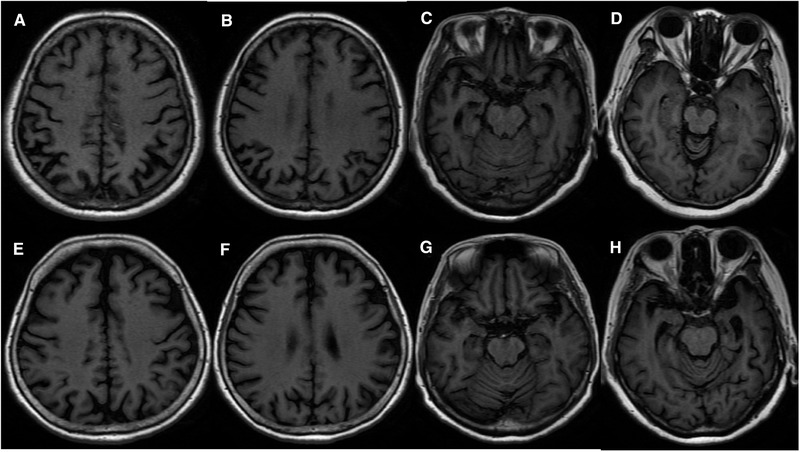
Brain MRI of 4 cases. A‐C, E‐G are from two female EOAD cases with a disease course of three years. The case with A‐C is 62 years old with ApoE genotype of ε3ε3, and the case with E‐G is 63 years old with ε4ε4. The ε3ε3 carrier shows greater cortical atrophy than the ε4ε4 carrier in parietal, occipital and temporal regions. D and H are from two female LOAD cases with a disease course of two years. They are 76 and 75 years old, respectively. ApoE genotype are ε2ε3 and ε3ε4, respectively. The ε3ε4 carrier shows greater cortical atrophy than the ε2ε3 carrier, mainly in medial temporal lobes

As illustrated in Figures 2 and 3, in LOAD, ɛ4 carriers demonstrated reduced cortical thickness (mm^3^) than ɛ4 non‐carriers in medial temporal areas, including the bilateral hippocampus (1809 ± 417 versus 2055 ± 3, *p*  = .013; 2132 ± 395 versus 2366 ± 412, *p*  = .009), bilateral parahippocampus (2066 ± 362 versus 2259 ± 335, *p*  = .016; 2127 ± 335 versus 2300 ± 323, *p*  = .019), and bilateral entorhinal regions (1423 ± 329 versus 1663 ± 316, *p*  = .004; 1517 ± 342 versus 1712 ± 328, *p*  = .018).

In EOAD, ɛ4 non‐carriers showed smaller cortical volume (mm^3^) than ɛ4 carriers in widespread areas, including left entorhinal (1665 ± 306 versus 1742 ± 315, *p*  = .036), bilateral middle cingulate (3083 ± 477 versus 3353 ± 426, *p*  = .026; 3277 ± 405 versus 3573 ± 504, *p*  = .004), right inferior frontal (2740 ± 375 versus 3021 ± 432, *p*  = .019), as well as left postcentral (7957 ± 1166 versus 8419 ± 1274, *p*  = .026), left angular (6325 ± 1493 versus 6786 ± 1488, *p*  = .046) and left calcarine regions (2891 ± 494 versus 3055 ± 565, *p*  = .038). Accordingly, the total CSF volume (mm^3^) was greater in ɛ4 non‐carriers relative to ɛ4‐carriers (617715 ± 131897 versus 524704 ± 123456, *p*  = .018).

### 3.5 CSF biomarker (Supporting Information 4)

No difference was found between EOAD and LOAD in CSF Aβ42, p‐tau, t‐tau levels, and p‐tau/Aβ42, t‐tau/Aβ42 ratios.

**FIGURE 4:**
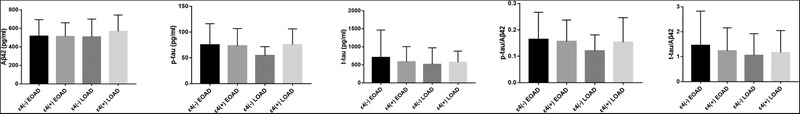
Comparison of CSF biological features between ε4 carriers and non‐carriers in both EOAD and LOAD. The data were compared by general linear model. Gender, age, and disease course were included in the model as fixed factor or covariate

As illustrated in Figure 4, in LOAD, ε4 carriers had a slightly higher Aβ42 (567.5 ± 176.0 versus 507.7 ± 192.4 mg/dl, *p*  = .412) and p‐tau level (75.7 ± 30.4 versus 54.8 ± 17.0 mg/dl, *p*  = .075) than non‐carriers.

In EOAD, ε4 carriers had a bit lower t‐tau (583.2 ± 421.3 versus 703.6 ± 760.9 mg/dl, *p*  = .366) and t‐tau/Aβ42 ratio (1.24 ± 0.92 versus 1.46 ± 1.37, *p*  = .361) than non‐carriers. However, these differences were not statistically significant.

### 3.6 Pathogenic mutation (Supporting Information 5)

Herein, we concentrated on the potential pathogenic variants in APP, PSEN1, and PSEN2. 6.2% (43/690) subjects harbored potential causative mutations. EOAD patients had a slightly higher mutation frequency than LOAD patients (7.1% versus 5.3%).

After stratification by ApoE‐ɛ4 status and AOO, the cohort was divided into four subgroups, ε4‐negative EOAD, ε4‐positive EOAD, ε4‐negative LOAD, and ε4‐positive LOAD patients. ε4‐negative EOAD cases showed the highest mutation frequency (7.8% versus 6.1%, 5.5%, 5.1%). However, these differences did not reach statistical significance.

## 4 DISCUSSION

### 4.1 Heterogeneity between EOAD and LOAD

As stated above, LOAD patients show a higher ApoE‐ɛ4 allele frequency and less occipital atrophy than EOAD patients. Is the phenotypic difference between EOAD and LOAD related to their difference in ApoE genotype?

After stratification by ApoE‐ɛ4 status, the imaging difference remains between ɛ4‐negative EOAD and LOAD patients. And cognitive difference appears between ɛ4‐positive EOAD and LOAD patients. These suggest that the phenotypic heterogeneity between EOAD and LOAD is not simply due to their difference in ApoE genotype.

This paper focuses on the effect of ApoE on clinical phenotypes of EOAD and LOAD, respectively.

### 4.2 Heterogeneous effect of ApoE‐ɛ4 in EOAD and LOAD

#### 4.2.1 ApoE‐ɛ4 and female predominance in LOAD

In LOAD, ɛ4‐carriers show female predominance relative to non‐carriers, which is consistent with the previous study (Riedel et al., 2016). ApoE‐ɛ4 is supposed to have a higher risk in women than men for developing AD (Ungar et al., 2014).

#### 4.2.2 ApoE‐ɛ4 and memory, executive deficit in EOAD, LOAD

In both EOAD and LOAD, ApoE‐ɛ4 is associated with the deterioration of memory and executive functions. The memory difference between ε4 carriers and non‐carriers is more obvious in LOAD. It can be found in both verbal and non‐verbal memory tests, such as AVLT and rey figure recall.

Both EOAD and LOAD patients retain a certain learning ability since the third immediate recall of AVLT has improved significantly relative to the first recall. However, this improvement does not differ by ApoE‐ɛ4 status, which suggests that ApoE‐ɛ4 might have little effect on learning ability.

In EOAD and LOAD, ε4 carriers show worse performances in modified Luria's tapping task and TMT‐A than non‐carriers, respectively. Both tests are commonly used in assessing executive function. In LOAD, ɛ4 carriers and non‐carriers differ in the error numbers of TMT‐A, but not in the task completion time. This suggests that ApoE‐ɛ4 might affect other executive resources rather than psychomotor speed.

#### 4.2.3 Inverse effect of ApoE‐ɛ4 on cortical thickness in EOAD, LOAD

In LOAD, ε4 carriers have smaller cortical volume than non‐carriers, mainly limited to medial temporal lobes. However, in EOAD, ε4 non‐carriers show greater cortical atrophy than ε4 carriers in widespread areas, including medial temporal, cingulate, inferior frontal and parieto‐occipital cortices, etc. The different atrophy patterns might be attributed to the intrinsic heterogeneity between EOAD and LOAD, as well as the heterogeneous effect of ApoE‐ε4 and non‐ε4 alleles.

In LOAD, the cognitive differences between ε4 carriers and non‐carriers might be explained by their cortical morphometric differences. Hippocampal, parahippocampal, and entorhinal areas are involved in the memory processing system. In addition, the hippocampal network is functionally linked with frontal, temporal, parietal, and occipital lobes. The decreased connections between the hippocampal network and the frontal/prefrontal cortex might be responsible for the worse executive function in ε4‐carriers relative to non‐carriers (Chand et al., 2018; Hartung et al., 2016; Schneider et al., 2019).

In EOAD, there is a contradictory finding between cognitive function and cortical volume. ε4 carriers have worse cognitive performance but less cortical atrophy than non‐carriers. The better cognitive performance of ε4 non‐carriers might be related to their greater CSF volume. Some studies demonstrate that the increased CSF volume is associated with inhibited Aβ aggregation and decreased tau level, probably due to the altered blood‐brain barrier (Ott et al., 2010; Padayachee et al., 2016).

#### 4.2.4 ApoE‐ɛ4 and tau burden in EOAD, LOAD

In LOAD, ε4 carriers show a bit higher p‐tau level than non‐carriers. In EOAD, ε4 carriers show a bit lower t‐tau than ε4 non‐carriers. The inverse effect of ApoE‐ε4 on tau burden in EOAD and LOAD is similar to its inverse effect on cortical volume, as discussed in 4.2.3. It is unclear whether ApoE‐ɛ4 plays a role in tau‐related pathways.

#### 4.2.5 ApoE‐ɛ4 and causative mutation in EOAD, LOAD

Mutation frequency does not differ by ApoE status. More than 90% of the whole AD cohort do not carry causative mutations in APP, PSEN1, and PSEN2. Further genetic research is expected, especially for ε4‐negative EOAD subjects.

ε4‐negative EOAD cases do not carry ɛ4 allele which is identified as a deleterious factor for AD. Nonetheless, they have severe and extensive cortical atrophy. There might be an extra‐strong genetic factor which initiates all the processes.

### 4.3 Conclusion and limitation

EOAD and LOAD are two heterogeneous entities. ApoE exerts a heterogeneous effect on their clinical phenotypes. As expected, in LOAD, ApoE‐ɛ4 genotype is associated with worse cognitive function and severe medial temporal atrophy. However, in EOAD, ɛ4 genotype is associated with worse cognitive function but less cortical atrophy in widespread areas. The whole heterogeneity might be related to their different underlying genetic and pathological basis.

The main limitation of this paper is the potential bias related to small sample size and experimental implementation. In order to minimize the selection bias, we use the general linear model in statistical analysis, with gender, age, disease course, and educational level as confounding factors. In addition, all the participants in this study were clinically diagnosed as probable AD without autopsy confirmation. Only a few subjects had brain FDG‐PET or CSF testing of Aβ42, p‐tau, t‐tau. Next, we expect to expand the sample size and continue the follow‐up studies, in combination with pathological and genetic research.

## CONFLICT OF INTEREST

The authors have no conflict of interest to report.

## AUTHOR CONTRIBUTIONS

Liling Dong and Jie Li contributed to acquisition, analysis, interpretation of the data, and draft of the work. Jing Gao contributed to acquisition, analysis, interpretation of the data, and conception, revision of the work. Caiyan Liu, Chenhui Mao, Jie Wang, Xinying Huang, Dan Lei, Shanshan Chu, Bo Hou, Feng Feng, Longze Sha, and Qi Xu contributed to acquisition of the data. All authors approved the submitted version.

### PEER REVIEW

The peer review history for this article is available at https://publons.com/publon/10.1002/brb3.2373

## Supporting information

Supporting Information

## Data Availability

The original contributions are included in the article; further datasets are available from the corresponding author on reasonable request.
