# Supplementary material for: Effects of ApoE genotype on clinical phenotypes in early‐onset and late‐onset Alzheimer's disease in China: Data from the PUMCH dementia cohort
Source: Brain Behav. 2021 Sep 23;11(11):e2373. doi: 10.1002/brb3.2373 (PMC8613405; doi:10.1002/brb3.2373)

**Supplement 1**

**Neuropsychological features (mean±SD) (screening test)**

|  |  | AD (n=785) | | | | |  | EOAD (n=386) | | | | |  | LOAD (n=399) | | | | |  |  | |
| --- | --- | --- | --- | --- | --- | --- | --- | --- | --- | --- | --- | --- | --- | --- | --- | --- | --- | --- | --- | --- | --- |
| Screening test |  | EOAD (n=386) | LOAD (n=399) | | P | |  | ε4 non-carrier (n=235) | | ε4 carrier (n=151) | | P |  | ε4 non-carrier (n=210) | | ε4 carrier (n=189) | P | |  | P^†^ | P^‡^ |
| MMSE |  | 16.8±8.9 | | 17.4±7.5 | | 0.561 |  | 17.3±9.0 | 16.1±8.7 | | 0.777 | |  | 18.1±7.6 | 16.7±7.4 | | | 0.475 |  | 0.796 | 0.549 |
| Moca |  | 13.4±8.4 | | 14.0±7.1 | | 0.933 |  | 13.8±8.7 | 12.8±8.0 | | 0.616 | |  | 14.6±7.3 | 13.4±6.8 | | | 0.837 |  | 0.847 | 0.794 |
| ADLs |  | 35.0±11.8 | | 36.2±11.5 | | 0.454 |  | 34.5±11.9 | 35.9±11.5 | | 0.780 | |  | 35.3±11.1 | 37.2±11.9 | | | 0.793 |  | 0.676 | 0.558 |
| HADS-A |  | 3.6±3.5 | | 2.9±3.2 | | 0.476 |  | 3.9±3.7 | 3.0±3.0 | | 0.475 | |  | 2.9±3.2 | 3.0±3.3 | | | 0.699 |  | 0.811 | 0.681 |
| HADS-D |  | 4.1±3.8 | | 3.9±3.8 | | 0.201 |  | 4.1±3.9 | 4.0±3.7 | | 0.566 | |  | 3.8±3.7 | 4.1±3.9 | | | 0.842 |  | 0.094 | 0.607 |

The data were compared by general linear model. Gender, age, disease course and educational level were included in the model as fixed factor or covariate.

P^†^, comparisons were made between ε4-negative EOAD and LOAD subjects; P^‡^, comparisons were made between ε4-positive EOAD and LOAD subjects.

EOAD, early-onset alzheimer’s disease; LOAD, late-onset alzheimer’s disease; MMSE, mini-mental state exam; Moca, Montreal cognitive assessment; ADL, activities of daily living; HADS-A, hospital anxiety and depression scale-anxiety subscale; HADS-D, hospital anxiety and depression scale-depression subscale.

**Supplement 2**

**Neuropsychological features (mean±SD) (domain assessment)**

|  |  | AD (n=317) | | |  | EOAD (n=142) | | |  | LOAD (n=175) | | |  |  |  |
| --- | --- | --- | --- | --- | --- | --- | --- | --- | --- | --- | --- | --- | --- | --- | --- |
| Domain assessment |  | EOAD  (n=142) | LOAD  (n=175) | P |  | ε4 non-carrier  (n=85) | ε4 carrier  (n=57) | P |  | ε4 non-carrier  (n=97) | ε4 carrier  (n=78) | P |  | P^†^ | P^‡^ |
| Word fluency |  | 15.1±6.6 | 14.1±5.4 | 0.162 |  | 15.3±7.1 | 14.7±5.8 | 0.066 |  | 14.5±5.3 | 13.6±5.4 | 0.183 |  | 0.129 | 0.526 |
| DSST |  | 24.9±15.5 | 23.8±11.6 | 0.971 |  | 25.7±15.5 | 23.7±15.5 | 0.132 |  | 23.7±10.5 | 23.8±12.9 | 0.846 |  | 0.645 | 0.690 |
| TMT-A (seconds)^§^ |  | 98.9±79.2  (n=107) | 120.1±65.7  (n=141) | 0.822 |  | 100.7±95.3  (n=65) | 96.2±45.1  (n=42) | 0.919 |  | 123.9±71.0  (n=85) | 114.4±56.9  (n=56) | 0.433 |  | 0.589 | 0.028**^*^** |
| TMT-A (errors) |  | 2.6±6.7 | 1.5±4.6 | 0.311 |  | 3.0±7.3 | 2.2±5.7 | 0.932 |  | 0.7±2.5 | 2.5±6.1 | 0.001**^***^** |  | 0.210 | 0.291 |
| Single gesture imitation |  | 5.4±2.0 | 5.8±1.6 | 0.290 |  | 5.5±1.9 | 5.4±3.1 | 0.312 |  | 5.7±1.6 | 5.9±1.6 | 0.395 |  | 0.721 | 0.317 |
| Modified Luria’s task |  | 1.3±1.2 | 1.3±1.1 | 0.404 |  | 1.5±1.2 | 1.1±1.1 | 0.020**^*^** |  | 1.3±1.1 | 1.2±1.0 | 0.696 |  | 0.749 | 0.740 |
| Clock drawing |  | 1.8±1.0 | 1.9±0.9 | 0.888 |  | 1.8±1.0 | 1.9±1.0 | 0.709 |  | 2.0±0.9 | 1.8±0.9 | 0.352 |  | 0.789 | 0.496 |
| Graphics copying |  | 7.6±3.2 | 8.7±2.2 | 0.336 |  | 7.5±3.2 | 7.6±3.2 | 0.624 |  | 8.7±2.2 | 8.7±2.2 | 0.829 |  | 0.504 | 0.209 |
| Block design test |  | 4.8±3.6 | 5.6±3.1 | 0.931 |  | 4.9±3.7 | 4.8±3.6 | 0.037**^*^** |  | 5.7±3.1 | 5.5±3.2 | 0.187 |  | 0.675 | 0.893 |
| Rey figure-Copy |  | 14.0±4.3  (n=114) | 15.3±1.4  (n=128) | 0.805 |  | 13.6±4.9  (n=74) | 14.6±3.1  (n=40) | 0.568 |  | 15.2±1.5  (n=72) | 15.5±1.2  (n-56) | 0.927 |  | 0.621 | 0.611 |
| Rey figure-Recall |  | 7.0±5.7 | 4.9±5.1 | 0.354 |  | 7.0±5.7 | 7.0±5.9 | 0.904 |  | 6.1±5.2 | 3.4±4.6 | 0.004**^**^** |  | 0.596 | 0.093 |
| AVLT-N1 |  | 3.1±1.7 | 2.8±1.5 | 0.684 |  | 3.2±1.8 | 2.9±1.7 | 0.115 |  | 2.9±1.5 | 2.6±1.4 | 0.104 |  | 0.586 | 0.667 |
| AVLT-N2 |  | 4.5±24 | 4.2±1.8 | 0.519 |  | 4.5±2.4 | 4.5±2.5 | 0.903 |  | 4.3±1.9 | 4.1±1.8 | 0.291 |  | 0.529 | 0.792 |
| AVLT-N3 |  | 5.1±2.5 | 4.8±2.1 | 0.497 |  | 5.3±2.5 | 4.9±2.5 | 0.160 |  | 4.9±2.2 | 4.7±2.0 | 0.464 |  | 0.834 | 0.444 |
| AVLT-I |  | 12.7±6.2 | 11.8±4.9 | 0.519 |  | 13.0±6.2 | 12.3±6.1 | 0.289 |  | 12.1±5.1 | 11.4±4.7 | 0.229 |  | 0.991 | 0.585 |
| AVLT-N4 |  | 3.1±3.1 | 1.9±2.5 | 0.897 |  | 3.7±3.0 | 2.2±3.0 | 0.014**^*^** |  | 2.3±2.7 | 1.3±2.2 | 0.014**^*^** |  | 0.694 | 0.986 |
| AVLT-N5 |  | 2.6±3.1 | 1.5±2.4 | 0.928 |  | 3.2±3.0 | 1.8±3.0 | 0.006**^**^** |  | 1.9±2.6 | 1.1±2.1 | 0.024**^*^** |  | 0.515 | 0.970 |
| Associate learning |  | 5.7±3.8 | 5.4±3.7 | 0.121 |  | 6.2±4.1 | 5.0±3.2 | 0.146 |  | 5.8±3.4 | 4.8±4.0 | 0.175 |  | 0.680 | 0.462 |
| Episodic memory |  | 5.2±4.0 | 4.5±3.3 | 0.664 |  | 5.7±4.1 | 4.4±3.6 | 0.041**^*^** |  | 5.0±3.4 | 3.8±3.2 | 0.065 |  | 0.661 | 0.764 |
| Similarity |  | 12.6±5.6 | 13.9±4.8 | 0.649 |  | 12.7±5.5 | 12.5±5.7 | 0.475 |  | 14.3±4.6 | 13.5±5.0 | 0.974 |  | 0.266 | 0.810 |
| Calculation |  | 8.1±4.1 | 8.4±3.4 | 0.696 |  | 8.1±4.2 | 8.1±4.0 | 0.470 |  | 8.2±3.4 | 8.8±3.4 | 0.423 |  | 0.842 | 0.931 |
| Oral comprehension |  | 7.6±0.7 | 7.5±0.7 | 0.254 |  | 7.5±0.8 | 7.6±0.7 | 0.194 |  | 7.5±0.7 | 7.5±0.7 | 0.993 |  | 0.379 | 0.219 |
| Repetition |  | 31.1±5.3 | 32.1±2.5 | 0.399 |  | 30.8±5.7 | 31.4±4.7 | 0.337 |  | 32.0±2.5 | 32.2±2.4 | 0.927 |  | 0.569 | 0.234 |
| Object naming |  | 9.7±0.9 | 9.9±0.4 | 0.971 |  | 9.6±1.1 | 9.9±0.5 | 0.267 |  | 9.9±0.4 | 9.9±0.4 | 0.195 |  | 0.410 | 0.287 |
| Colour naming |  | 5.8±0.6 | 6.0±01 | 0.821 |  | 5.8±0.7 | 5.9±0.4 | 0.144 |  | 6.0±0.1 | 6.0±0.2 | 0.552 |  | 0.291 | 0.617 |
| Spontaneous speech  (fluent/intermediate/non-fluent) |  | 106/21/15 | 135/15/25 | 0.166 |  | 63/13/9 | 43/8/6 | 0.978 |  | 73/10/14 | 62/5/11 | 0.647 |  | 0.490 | 0.302 |

The data were compared by general linear model. Gender, age, disease course and educational level were included in the model as fixed factor or covariate.

P^†^, comparisons were made between ε4-negative EOAD and LOAD subjects; P^‡^, comparisons were made between ε4-positive EOAD and LOAD subjects.

TMT-A^§^, the subjects with more than one error in TMT-A was excluded from the statistical analysis of task completion time.

EOAD, early-onset alzheimer’s disease; LOAD, late-onset alzheimer’s disease; DSST, digital symbol substitution task; TMT-A, trail making test part A; AVLT-N1, AVLT-N2, AVLT-N3 and AVLT-I, the first, second, third and total immediate recall of auditory verbal learning test; AVLT-N4 and AVLT-N5, short an long delayed recall of AVLT.

**Supplement 3**

**MRI morphometric features (mean±SD) (mm^3^)**

|  |  | AD (n=130) | | |  | EOAD (n=48) | | |  | LOAD (n=82) | | |  |  | |
| --- | --- | --- | --- | --- | --- | --- | --- | --- | --- | --- | --- | --- | --- | --- | --- |
|  |  | EOAD  (n=48) | LOAD  (n=82) | P |  | ε4 noncarrier  (n=21) | ε4 carrier  (n=27) | P |  | ε4noncarrier  (n=40) | ε4 carrier  (n=42) | P |  | P^†^ | P^‡^ |
| Total Incranial Volume |  | 1548766±134568 | 1507119±137873 | 0.096 |  | 1587596±140289 | 1518565±124219 | 0.078 |  | 1539137±113827 | 1476624±152569 | 0.039**^*^** |  | 0.150 | 0.236 |
| Total Grey Matter |  | 531281±58049 | 522421±49527 | 0.576 |  | 522055±55536 | 538457±59972 | 0.025**^*^** |  | 528490±49647 | 516642±49308 | 0.663 |  | 0.161 | 0.514 |
| Total White Matter |  | 449013±42968 | 416231±48089 | 0.622 |  | 444646±35222 | 452409±48536 | 0.041**^*^** |  | 412882±44455 | 419420±51648 | 0.067 |  | 0.091 | 0.465 |
| Total Cerebrospinal Fluid |  | 565396±134196 | 557674±101234 | 0.508 |  | 617715±131897 | 524704±123456 | 0.018**^*^** |  | 583584±77777 | 532998±114952 | 0.607 |  | 0.043**^*^** | 0.460 |
| Left Hippocampus |  | 2120±426 | 1929±410 | 0.849 |  | 2155±385 | 2093±460 | 0.713 |  | 2055±367 | 1809±417 | 0.013**^*^** |  | 0.392 | 0.253 |
| Right Hippocampus |  | 2447±513 | 2246±418 | 0.923 |  | 2491±461 | 2413±556 | 0.723 |  | 2366±412 | 2132±395 | 0.009**^**^** |  | 0.939 | 0.737 |
| Left Parahippocampus |  | 2411±382 | 2160±360 | 0.953 |  | 2380±344 | 2435±414 | 0.057 |  | 2259±335 | 2066±362 | 0.016**^*^** |  | 0.306 | 0.160 |
| Right Parahippocampus |  | 2370±359 | 2211±338 | 0.284 |  | 2390±321 | 2354±391 | 0.349 |  | 2300±323 | 2127±335 | 0.019**^*^** |  | 0.513 | 0.567 |
| Left Entorhinal Area |  | 1709±310 | 1540±343 | 0.669 |  | 1665±306 | 1742±315 | 0.036**^*^** |  | 1663±316 | 1423±329 | 0.004**^**^** |  | 0.584 | 0.131 |
| Right Entorhinal Area |  | 1751±345 | 1612±347 | 0.868 |  | 1730±313 | 1767±374 | 0.158 |  | 1712±328 | 1517±342 | 0.018**^*^** |  | 0.734 | 0.904 |
| Left Middle Cingulate |  | 3235±464 | 3192±426 | 0.801 |  | 3083±477 | 3353±426 | 0.026**^*^** |  | 3237±448 | 3149±405 | 0.758 |  | 0.384 | 0.239 |
| Right Middle Cingulate |  | 3444±482 | 3425±477 | 0.400 |  | 3277±405 | 3573±504 | 0.004**^**^** |  | 3470±501 | 3382±455 | 0.774 |  | 0.543 | 0.361 |
| Left Posterior Cingulate |  | 3036±519 | 3176±438 | 0.883 |  | 2990±456 | 3071±569 | 0.138 |  | 3276±438 | 3080±421 | 0.338 |  | 0.043**^*^** | 0.198 |
| Right Posterior Cingulate |  | 2812±484 | 2925±412 | 0.799 |  | 2778±480 | 2839±495 | 0.148 |  | 2996±436 | 2857±380 | 0.493 |  | 0.491 | 0.831 |
| Left Inferior Frontal |  | 2876±464 | 2747±402 | 0.188 |  | 2777±468 | 2954±454 | 0.095 |  | 2768±399 | 2727±409 | 0.965 |  | 0.695 | 0.108 |
| Right Inferior Frontal |  | 2898±428 | 2757±403 | 0.902 |  | 2740±375 | 3021±432 | 0.019**^*^** |  | 2778±422 | 2737±388 | 0.386 |  | 0.613 | 0.510 |
| Left Postcentral |  | 8217±1237 | 7750±1064 | 0.258 |  | 7957±1166 | 8419±1274 | 0.026**^*^** |  | 7813±974 | 7689±1151 | 0.451 |  | 0.108 | 0.983 |
| Right Postcentral |  | 7451±1181 | 7002±1013 | 0.269 |  | 7323±1121 | 7550±1237 | 0.196 |  | 6925±889 | 7076±1124 | 0.789 |  | 0.589 | 0.534 |
| Left Superior Parietal Lobule |  | 7808±1572 | 8216±1040 | 0.160 |  | 7745±1483 | 7857±1665 | 0.344 |  | 8174±1088 | 8255±1004 | 0.267 |  | 0.035**^*^** | 0.830 |
| Right Superior Parietal Lobule |  | 7728±1374 | 8246±955 | 0.544 |  | 7770±1180 | 7695±1530 | 0.483 |  | 8381±976 | 8117±927 | 0.536 |  | 0.516 | 0.971 |
| Left Angular |  | 6584±1492 | 7246±1087 | 0.086 |  | 6325±1493 | 6786±1488 | 0.046**^*^** |  | 7377±973 | 7121±1184 | 0.814 |  | 0.015**^*^** | 0.898 |
| Right Angular |  | 7909±2001 | 8682±1192 | 0.287 |  | 7649±2020 | 8112±2001 | 0.110 |  | 8885±1266 | 8489±1097 | 0.263 |  | 0.086 | 0.900 |
| Left Middle Occipital |  | 4572±1057 | 4818±673 | 0.020**^*^** |  | 4549±1139 | 4589±1010 | 0.282 |  | 4900±709 | 4740±636 | 0.400 |  | 0.005**^**^** | 0.552 |
| Right Middle Occipital |  | 3485±795 | 3776±508 | 0.210 |  | 3536±856 | 3445±759 | 0.560 |  | 3846±493 | 3709±519 | 0.370 |  | 0.065 | 0.889 |
| Left Inferior Occipital |  | 4604±1047 | 4865±806 | 0.023**^*^** |  | 4546±938 | 4650±1140 | 0.134 |  | 5016±845 | 4722±749 | 0.190 |  | 0.003**^**^** | 0.634 |
| Right Inferior Occipital |  | 4926±1089 | 5104±672 | 0.099 |  | 5134±1069 | 4764±1097 | 0.907 |  | 5154±724 | 5057±624 | 0.963 |  | 0.416 | 0.191 |
| Left Occipital Pole |  | 2380±564 | 2229±535 | 0.072 |  | 2416±449 | 2352±646 | 0.404 |  | 2324±530 | 2139±531 | 0.512 |  | 0.011**^*^** | 0.650 |
| Right Occipital Pole |  | 2092±503 | 2025±506 | 0.125 |  | 2235±460 | 1981±515 | 0.440 |  | 2117±551 | 1938±450 | 0.384 |  | 0.431 | 0.188 |
| Left Occipital Fusiform |  | 2649±479 | 2638±371 | 0.023**^*^** |  | 2659±424 | 2642±526 | 0.246 |  | 2683±301 | 2595±427 | 0.491 |  | 0.002**^**^** | 0.527 |
| Right Occipital Fusiform |  | 2743±475 | 2712±411 | 0.058 |  | 2756±483 | 2733±477 | 0.206 |  | 2724±379 | 2700±444 | 0.739 |  | 0.172 | 0.318 |
| Left Calcarine |  | 2983±536 | 2652±534 | 0.159 |  | 2891±494 | 3055±565 | 0.038**^*^** |  | 2632±587 | 2671±483 | 0.474 |  | 0.390 | 0.381 |
| Right Calcarine |  | 3022±582 | 2630±492 | 0.633 |  | 3015±536 | 3028±626 | 0.454 |  | 2546±507 | 2709±470 | 0.118 |  | 0.574 | 0.466 |

The data were compared by general linear model. Gender, age, disease course and total intracranial volume were included in the model as fixed factor or covariate.

P^†^, comparisons were made between ε4-negative EOAD and LOAD subjects; P^‡^, comparisons were made between ε4-positive EOAD and LOAD subjects.

EOAD, early-onset alzheimer’s disease; LOAD, late-onset alzheimer’s disease.

**Supplement 4**

**CSF biological features (mean±SD)**

|  |  | AD (n=144) | | |  | EOAD (n=115) | | |  | LOAD (n=29) | | |  |  | |
| --- | --- | --- | --- | --- | --- | --- | --- | --- | --- | --- | --- | --- | --- | --- | --- |
|  |  | EOAD (n=115) | LOAD (n=29) | P |  | ε4 non-carrier (n=70) | ε4 carrier (n=45) | P |  | ε4 non-carrier (n=20) | ε4 carrier (n=9) | P |  | P^†^ | P^‡^ |
| Aβ42 (pg/ml) |  | 514.5±166.0 | 526.3±186.4 | 0.933 |  | 516.6±176.9 | 511.1±149.3 | 0.813 |  | 507.7±192.4 | 567.5±176.0 | 0.412 |  | 0.700 | 0.543 |
| p-tau (pg/ml) |  | 74.5±38.1 | 61.3±23.6 | 0.827 |  | 75.2±41.1 | 73.3±33.5 | 0.792 |  | 54.8±17.0 | 75.7±30.4 | 0.075 |  | 0.405 | 0.582 |
| t-tau (pg/ml) |  | 656.5±650.0 | 532.4±411.8 | 0.604 |  | 703.6±760.9 | 583.2±421.3 | 0.366 |  | 516.0±456.8 | 568.8±309.5 | 0.704 |  | 0.653 | 0.910 |
| p-tau/Aβ42 |  | 0.16±0.09 | 0.13±0.07 | 0.803 |  | 0.16±0.10 | 0.16±0.08 | 0.639 |  | 0.12±0.06 | 0.15±0.09 | 0.289 |  | 0.854 | 0.799 |
| t-tau/Aβ42 |  | 1.37±1.21 | 1.09±0.85 | 0.564 |  | 1.46±1.37 | 1.24±0.92 | 0.361 |  | 1.06±0.86 | 1.16±0.88 | 0.774 |  | 0.440 | 0.645 |

The data were compared by general linear model. Gender, age and disease course were included in the model as fixed factor or covariate.

P^†^, comparisons were made between ε4-negative EOAD and LOAD subjects; P^‡^, comparisons were made between ε4-positive EOAD and LOAD subjects.

EOAD, early-onset alzheimer’s disease; LOAD, late-onset alzheimer’s disease.

**Supplement 5**

**Genetic features**

|  |  | AD (n=690) | | |  | EOAD (n=351) | | |  | LOAD (n=339) | | |  |  | |
| --- | --- | --- | --- | --- | --- | --- | --- | --- | --- | --- | --- | --- | --- | --- | --- |
|  |  | EOAD (n=351) | LOAD (n=339) | P |  | ε4 non-carrier (n=219) | ε4 carrier (n=132) | P |  | ε4 non-carrier (n=182) | ε4 carrier (n=157) | P |  | P^†^ | P^‡^ |
| Pathogenic mutation (+/-) |  | 25/326 | 18/321 | 0.325 |  | 17/202 | 8/124 | 0.548 |  | 10/172 | 8/149 | 0.870 |  | 0.367 | 0.721 |
| APP/PSEN1/PSEN2 |  | 10/8/7 | 9/4/5 | 0.742 |  | 7/7/3 | 3/1/4 | 0.190 |  | 5/3/2 | 4/1/3 | 0.824 |  | 0.874 | 1.000 |

P^†^, comparisons were made between ε4-negative EOAD and LOAD subjects; P^‡^, comparisons were made between ε4-positive EOAD and LOAD subjects.

EOAD, early-onset alzheimer’s disease; LOAD, late-onset alzheimer’s disease.


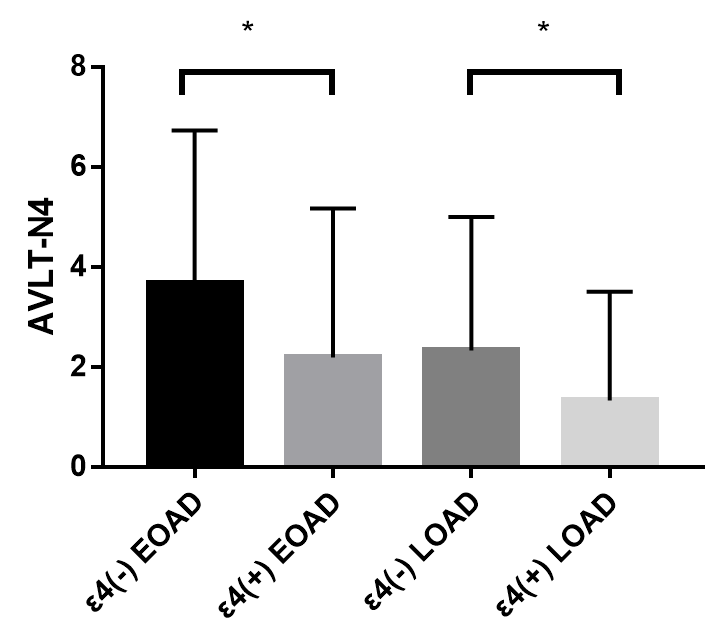

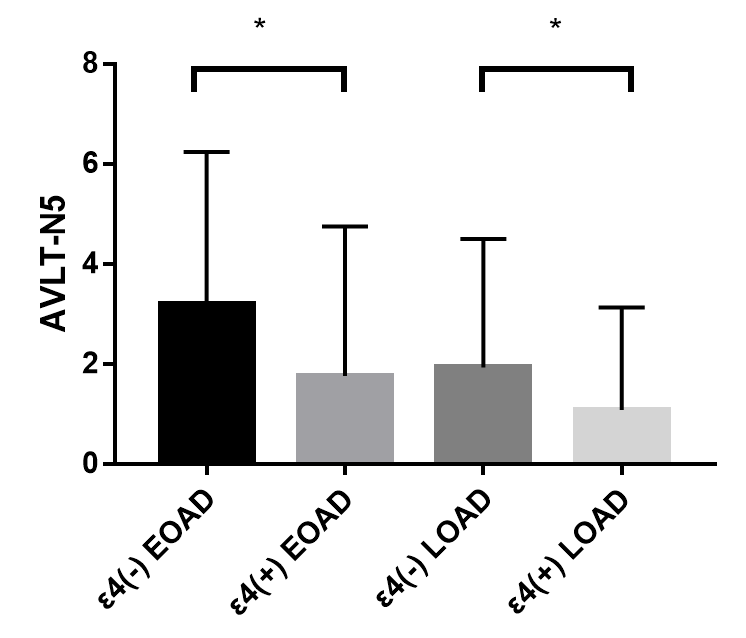

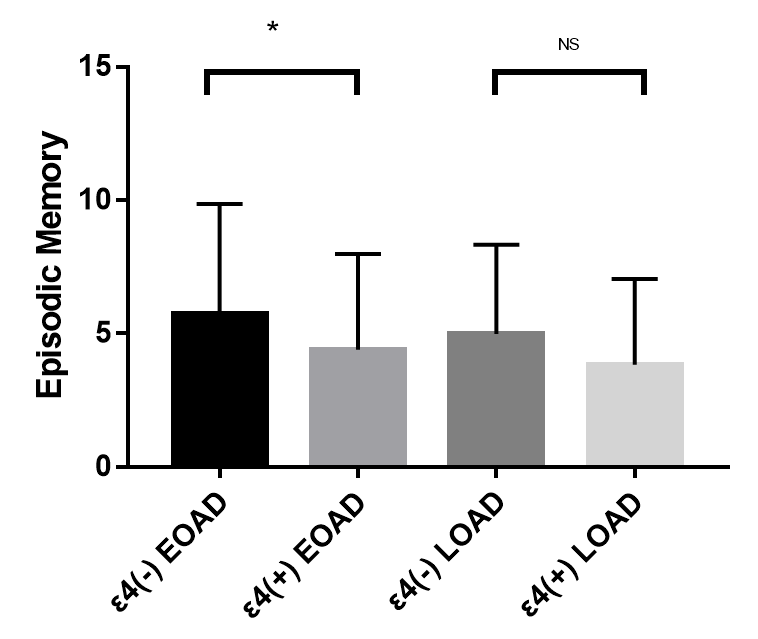

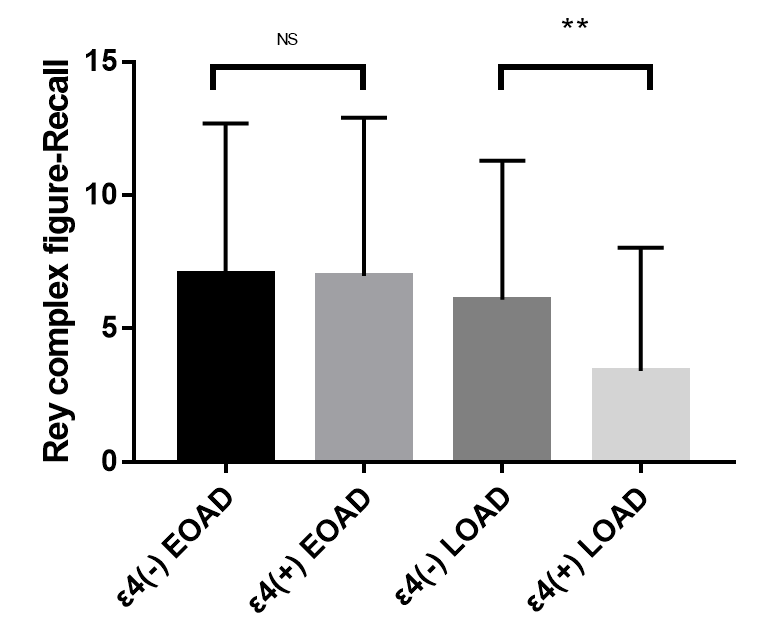

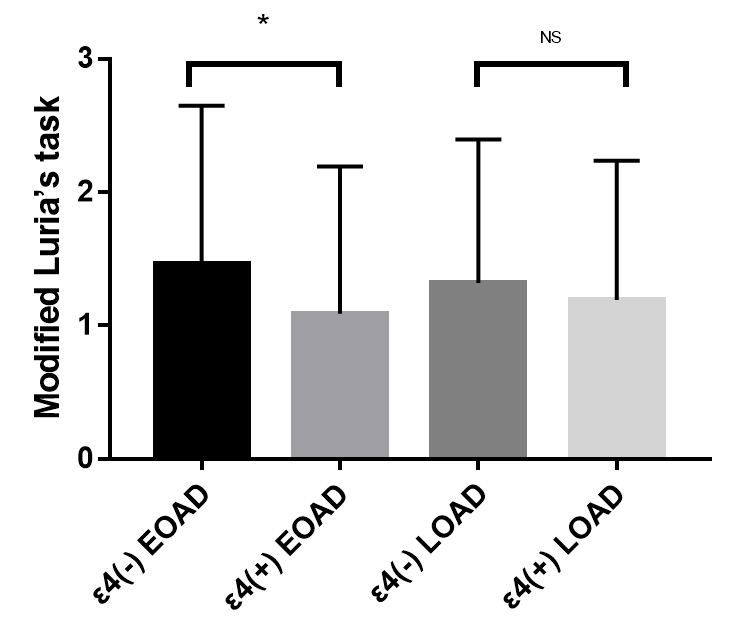

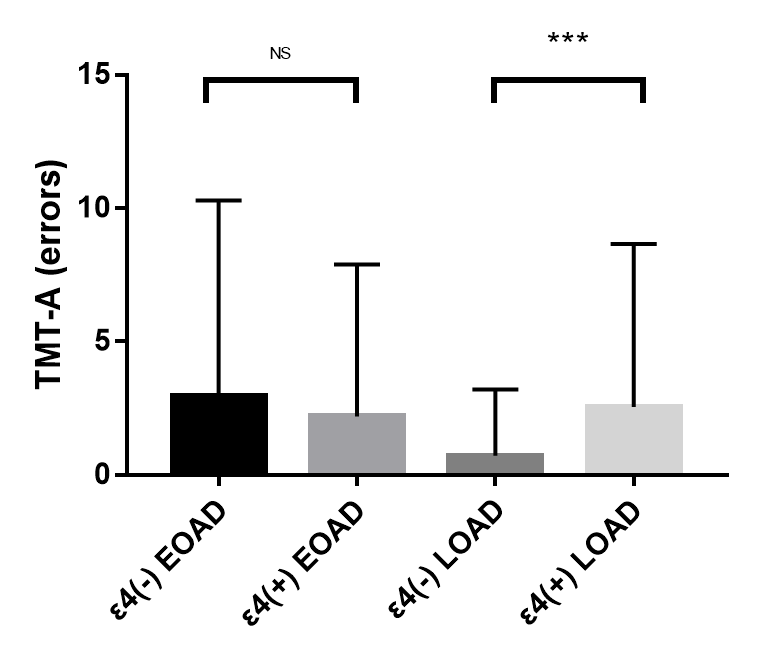

Supplement: Supplementary file 1 — Supporting Information [file BRB3-11-e2373-s001.docx]
